# Loneliness, student engagement, and academic achievement during emergency remote teaching during COVID-19: the role of the God locus of control

**DOI:** 10.1057/s41599-022-01328-9

**Published:** 2022-09-09

**Authors:** Hilmi Mizani, Ani Cahyadi, Hendryadi Hendryadi, Salamah Salamah, Santi Retno Sari

**Affiliations:** 1 Universitas Islam Negeri Antasari, Banjarmasin, Kalimantan Selatan Indonesia; 2 Sekolah Tinggi Ilmu Ekonomi Indonesia Jakarta, Jakarta, Indonesia; 3 Universitas Nasional, Jakarta, Indonesia

**Keywords:** Education, Philosophy

## Abstract

The COVID-19 pandemic has raised many problems in the education sector, one of which is the increasing trend toward student loneliness owing to a lack of interpersonal connections in online learning activities. The present study explicitly aims to examine the relationship between loneliness and academic achievement for university students in Indonesia. Moreover, we propose moderating God’s locus of control (i.e., God’s control over behavior-related learning) (GLC) and learning student engagement, playing mediating roles in these relationships. The data were collected from 324 respondents among university students in Indonesia during emergency remote teaching. The moderated-mediated regression analysis using Hayes’ PROCESS macro found loneliness negatively related to engagement and academic achievement. Student engagement had a positive relationship with academic achievement and served as a mediator between loneliness and academic achievement. Furthermore, GLC was found to moderate the relationship between loneliness and learning engagement as well as loneliness and academic achievement. This study’s findings uncover GLC’s role as a boundary condition, and confirms that learning-engagement intermediates the relationship between loneliness and academic achievement. Students with high perceived God control tend to anticipate the impact of loneliness on learning behavior amid isolation and loneliness because of the pandemic.

## Introduction

The COVID-19 pandemic has caused educational administrators to move face-to-face learning to an online environment to prevent virus transmission since the beginning of 2020. Almost all countries have problems with remote teaching (e.g., limited internet access and adjustment of materials), as learning modes that use online media are much more complex than conventional methods. Following the examples of schools in other countries that experienced significant challenges at the pandemic’s commencement, after 1 year, Indonesia’s educational institutions began adapting their policies to accommodate the situation (Cahyadi et al., 2021). Despite the various problems (e.g., connectivity issues and technological barriers), there was also an increasing trend towards a ‘crisis of connection’ and a ‘loneliness epidemic’ (Kaufmann and Vallade, 2020) associated with online learning. The long periods without face-to-face classroom interactions have led to concerns about students’ long-term psychological development.

Information technology has facilitated access to communication allowing people to connect from various regions. Interestingly, this relationship results in the ‘illusion of friendship without friendship’ (Wood and Turkle, 2012), which in turn causes students to feel lonely. Interactions created through online channels cannot form effective relational relationships because of the lack of interpersonal connections (Driver, 2018). Loneliness is an effective predictor of mental health and well-being (Diehl et al., 2021; Dinu et al., 2022; Kaufmann and Vallade, 2020; McIntyre-Mills et al., 2018; Wright, 2014), student engagement (Stoliker and Lafreniere, 2015), academic performance (Fan et al., 2021; Singh et al., 2021; Yalçın et al., 2020), lack of sense of community (Reedy, 2019), and dropout intentions (Alkan, 2014). Thus, it is necessary to explore the possible effect of loneliness on student learning experiences (Kaufmann and Vallade, 2020), such as student engagement and academic achievement.

Existing studies have confirmed the relationship between loneliness, engagement, and academic achievement, but there are limited remedies. First, previous studies generally associate loneliness with various student learning behaviors (e.g., student engagement, achievement, sense of community, dropout intentions, mental health, and well-being). However, no studies have discussed the model of the relationship between loneliness, student engagement, and achievement. Thus, this study uncovers student engagement’s role as a unique psychological mechanism mediating the effect of loneliness on academic achievement.

Second, we enrich our understanding of loneliness and its negative effects by incorporating the concept of the God locus (Welton et al., 1996) as another form of locus of control (LoC) (Rotter, 1966). As a personality trait associated with a person’s belief in God’s power controlling their lives, the God locus of control (GLC) still raises debate regarding its role in influencing individual behavior. Researchers (e.g., Welton et al., 1996; Zarzycka et al., 2021) link the GLC with external locus, thereby weakening achievement motivation and engagement. However, researchers believe that the GLC is related to the internal locus, which is more closely related to self-efficacy, motivation, and determination. This study broadens the understanding of the complex role of the GLC as an internal and external locus on student learning activities during the COVID-19 pandemic. Using the evolutionary theory of loneliness (ETL) (Cacioppo and Cacioppo, 2018) and positive and negative learning (PNL model) (Dormann et al., 2018), we placed the GLC as a moderator in the relationship between loneliness, engagement, and academic achievement using the background of students in non-Western countries.

Third, the present study enriches empirical evidence regarding the GLC in Asian countries, while previous studies have primarily concerned Western countries (Boyd and Wilcox, 2020a, 2020b; Murray et al., 2006; Price and Collett, 2012; Zarzycka et al., 2021). Furthermore, studies on the GLC have focused more on the health sector (e.g., Boyd and Wilcox, 2020a; Green and Hina, 2022) than the education sector—the focus here. Specifically, we expand and support recent empirical evidence concerning the effect of the GLC in an online learning setting during the pandemic.

## Theoretical framework and hypotheses

This study used the Cacioppo Evolutionary Theory of Loneliness (ETL) (Cacioppo and Cacioppo, 2018) and the positive and negative learning (PNL) model (Dormann et al., 2018). According to the ETL, the loneliness stemming from the perception of being socially isolated has unique consequences on individuals’ social behaviors. Lonely individuals are often motivated to engage in various social activities to reduce feelings of loneliness and protect themselves against possible threats (Keller et al., 2022). However, loneliness also diminishes people’s motivation to engage in social interactions (Cacioppo and Cacioppo, 2018, p. 14) if their previous experiences did not achieve their goals. Cacioppo’s ETL illustrates that the consequences of loneliness are closely related to individuals’ prior experiences in social interactions; the unfulfilled need for social relationships can create the perception of being lonely. Several recent studies used ETL as a theoretical framework in the COVID-19 context (e.g., Hutten et al., 2021; Keller et al., 2022; Saporta et al., 2021; Wang et al., 2020) to explain the pandemic’s association with distress, mental health, and loneliness. This study’s participants might have experienced loneliness when the government policies limited social interactions and promoted self-isolation to reduce COVID-19’s spread. Thus, we used loneliness as a starting point and focused on its consequences for students’ learning behaviors (engagement and achievement).

Next, we integrated the PNL model with loneliness and the GLC as resources in learning engagement. The PNL model is a modified job-demands resource model (JD-R model) (Bakker and Demerouti, 2007; Schaufeli et al., 2002) that explains forming work engagements and burnout in the work environment. In line with the JD-R model, the PNL model describes two sources of learning-engagement formation, including study-related demands (e.g., learning tasks, time pressure, self-control demands) and learning and personal resources (e.g., feedback, social support from teachers and parents, technology support, self-efficacy), which affect students’ learning activities. The PNL model also explains that learning-engagement has consequences for critical thinking. The consequences of learning engagement for learning achievement must be explored in our comprehensive view of the PNL model. The PNL model also aligns with the JD-R model, which describes various positive consequences of work engagement (e.g., in-role and extra-role performance, creativity, OCB).

### Loneliness and student engagement

Loneliness is a concept that describes a discrepancy in the quality of the network of expected and received relationships (Peplau and Caldwell, 1978; Perlman, 2004). Loneliness is also a negative feeling caused by deficits in social relations (Dykstra and Fokkema, 2007). Therefore, it is always associated with an individual’s personal and social resources and restrictions (de Gierveld, 1998). In the education context, loneliness is associated with a lack of social presence and interaction (Kaufmann and Vallade, 2020; Kim et al., 2019) and is an issue of grave concern to researchers along with the use of online learning modes in the current decade. Thus, it is necessary to examine possible ways to reduce the perception of loneliness, especially given its negative impacts on student learning experiences (Kaufmann and Vallade, 2020).

Student engagement in the context of online learning may be more complex and ambiguous (Cole et al., 2021; Cole, 2018) than conventional face-to-face learning. Online learning’s characteristics that do not provide verbal interaction make it difficult for teachers to measure student engagement and respond accordingly (Cole et al., 2021). However, an established concept of engagement can refer to the definition proposed by Schaufeli et al. (2002), interpreted as a continuous and positive emotional state during the time of learning (Zhao et al., 2021). More specifically, engagement is a combination of emotional, cognitive, and behavioral components (Fredricks et al., 2004). Each component has a role: First, the emotional component provides an overview of students’ positive or negative attitudes regarding their learning activities. These attitudes are formed based on he/she perceptions, especially their relationships with fellow students, teachers, and institutions. The cognitive component refers to students’ attitudes towards the relevance of academic work and learning methods that use a cognitive approach to trigger their creativity in solving problems and self-regulated learning (Wang and Eccles, 2012). Finally, the behavioral component refers to how students behave in schools, such as their attendance and active participation, task assignment, and persistence in learning (Dormann et al., 2018; Finn and Zimmer, 2012; Singh et al., 2021).

Drawing on the PNL model (Dormann et al., 2018), loneliness is a negative personal resource confirmed to have deleterious effects on emotions, including mental health and well-being (Diehl et al., 2021; Dinu et al., 2022; Heredia et al., 2017; Kaufmann and Vallade, 2020; Wright, 2014). Since learning engagement is a combination of emotional, cognitive, and behavioral components (Dormann et al., 2018; Fredricks et al., 2004), the relationship between loneliness and engagement becomes relevant. Loneliness has also been empirically confirmed to be related to student engagement (Stoliker and Lafreniere, 2015) and academic performance (Benner, 2011; Fan et al., 2021; Yalçın et al., 2020), and predictors of dropout intentions (Alkan, 2014). Loneliness results in students’ tendency to withdraw from class (Reedy, 2019). Similarly, Singh et al. (2021) investigated the role of loneliness on student engagement in India; their findings show that burnout causes loneliness, decreasing student engagement. In summary, loneliness is commonly found to be negatively related to engagement and academic achievement. We hypothesize that:

**H1**. Loneliness is negatively associated with student engagement

**H2**. Loneliness is negatively associated with academic achievement

During the COVID-19 pandemic, researchers reported decreased student engagement during online classes (Chen et al., 2020), which triggered a high rate of failure and dropout at the university level (Dyment et al., 2020). Efforts to maintain a high level of student learning engagement are essential for student achievement (Lei et al., 2018; Yoon et al., 2020). Moreover, the literature has documented learning engagement’s vital role as an antecedent of student academic achievement (Bergdahl et al., 2020; Eccles and Wang, 2012; Li et al., 2019; Pursel et al., 2016; Yoon et al., 2018). Based on previous research, we propose the following hypothesis:

**H3**. Student engagement is positively associated with academic achievement

Furthermore, we posit that loneliness influences academic achievement via a student engagement based on the JD-R model and the theoretical exposition concerning H1 and H3. In other words, loneliness leads to lower learning engagement. The engagement has a positive effect on academic achievement (Bergdahl et al., 2020; Eccles and Wang, 2012; Li et al., 2019; Pursel et al., 2016; Yoon et al., 2018), that is, higher learning engagement encourages the intrinsic motivation for academic achievements. In this process, student engagement serves as a unique psychological mechanism mediating the effect of loneliness on academic achievement. Based on the previous explanation, we argue that loneliness negatively impacts engagement. However, the continued relationship between loneliness and educational achievement will depend on engagement, which positively affects academic achievement. Therefore, we propose the following hypothesis.

**H4**: Student engagement mediates the relationship between loneliness and academic achievement.

### The God locus of control as a moderator

The concept of the GLC is derived from the LoC, which distinguishes a person’s belief in outcomes based on internal or external control. LoC was first introduced by Rotter (1966) and came from the social learning theory of personality. In simpler terms, Rotter explains that internal or external control is a continuum, and does not stand alone. Thus, someone will be declared to have internal or external tendencies based on their beliefs about how strongly their actions affect the results. People who tend to believe in their strength to obtain the expected results are a typical internal locus; on the contrary, individuals with external tendencies believe that what happens in their lives comes from external forces’ intervention (Jacobs-Lawson et al., 2011).

The GLC’s position can be linked as an internal or external locus. The first group stated that the GLC is associated with an external locus pioneered by Welton et al. (1996). More recent studies support this argument (e.g., Zarzycka et al., 2021). For example, Boyd and Wilcox (2020a, 2020b) found that the God locus of health control is more closely related to external LoC. In the context of student learning behavior, Zarzycka et al. (2021) proved that the perceived God control is consequential in increasing student procrastination. The second group stated that the GLC was more closely related to internal control, such as self-efficacy, motivation, and determination (Boyd and Wilcox, 2020a, 2020b). Thus, people with internal LoC possess high achievement motivation and engagement (Albert and Dahling, 2016; Cahyadi et al., 2021; Chukwuorji et al., 2018; Yang et al., 2017). Different results were reported by Ye and Lin (2015), who found a positive relationship between the internal LoC with loneliness and preference for online social interaction and a negative association with subjective well-being.

**Fig. 1:**
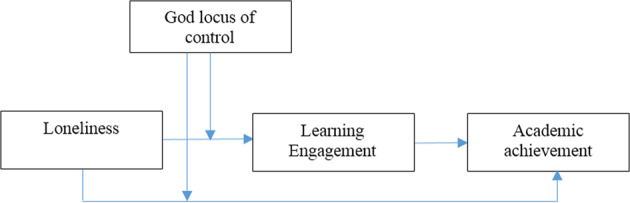
Research model. A description of the relationship between variables in the present study.

This study proposed that the GLC is a moderating variable in the relationship between loneliness, engagement, and academic achievement. Our approach improves previous studies that have examined loneliness as a predictor of engagement and student achievement without considering its possible role as a boundary condition. Thus, this study determined when and how personal resources (e.g., loneliness) affected student engagement and academic achievement. Based on the following arguments, we posited the GLC as a moderator of these relationships (see Fig. 1). Following the PNL model, we argued that loneliness is a personal response to social interactions and might negatively affect engagement and academic achievement; however, the GLC moderates the relationship’s strength as an external source. Similarly, the God locus can also be positioned as a positive religious coping behavior (Pargament, 2011) that neutralizes the destructive effects of stressful situations. Accordingly, we posited that the GLC is a positive religious coping behavior (Pargament, 2011) that is likely to be negatively correlated with loneliness and reduce its adverse effects on student engagement and achievement.

***H5***. The GLC moderates the effect of loneliness on student engagement.

***H6***. The GLC moderates the effect of loneliness on academic achievement.

## Methodology

### Sample and procedure

This study used a mixed purposive and snowball sampling method to determine the target sample. The primary researcher used personal communication with colleagues who were online course instructors at various universities to assist the data collection process. An online questionnaire designed with Google Forms was sent via WhatsApp groups or email to consenting collaborators. The questionnaire was only accessed by students from lecturers who had received approval to participate in the survey. Respondents’ participation was completely consensual, anonymous, and voluntary. Written informed consent from each participating university could not be received as names of respondents and names of universities were not included in the questionnaire.

Data were collected at two stages: at the beginning and the end of the semester. In the first phase, respondents were asked to provide biographical (gender, age, and grade), loneliness, and learning-engagement information. The first phase was carried out in March and April 2021, and received responses from 478 participants. In the second phase, participants who filled out the online questionnaire were invited via email to provide their views on GLC and learning achievement. The second phase was carried out from June to August 2021, and 343 participants (72 percent of the first phase) responded. After the elimination of unqualified data (duplication, partially filled questionnaires), the final data set was 324. As shown in Table 1, 64.2 percent of the respondents were female, the majority were bachelor’s degree holders (72.53 percent), and 53.40 percent of respondents were under 25 years (see Table 1).

**Table 1:** Characteristics of respondents.

Variable	Classification	Respondents	
		*N*	%
Gender	Male	116	35.80
	Female	208	64.20
Age	25 years old or below	173	53.40
	26–30 years old	89	27.47
	>30 years old	62	19.14
Degree	Bachelor	235	72.53
	Master	89	27.47

### Measurement

All scales used here were adapted from previous studies. To adapt items for Indonesia’s cultural context, we employed back translation through the expertize of a professional translator and two education experts with good English qualifications. Respondents were asked to give a rating based on a five-point Likert scale based on context, ranging from 1 (totally disagree/never) to 5 (totally agree/always). First, loneliness was measured using an eight-item short-scale (Hays and DiMatteo, 1987). Examples of items are, ‘I lack companionship’ and ‘people are around me, but not with me’. Cronbach’s *α* for this scale was 0.92, meeting the criteria for excellent internal consistency (Nunnally and Bernstein, 1994).

Second, we used the nine-item Utrecht Work Engagement Scale (UWES-9) developed by Schaufeli et al. (2002) to measure student engagement. We made minor changes to suit the context. Following previous studies (e.g., Cahyadi et al., 2021; Zhao et al., 2021), we replaced the word ‘workplace’ with ‘online class’ to describe engagement in classroom learning settings. The Cronbach’s *α* for the scale was 0.93.

The GLC was assessed through the 6-item God Locus of Health Control scale (GLHC, Wallston et al., 1999) to measure the influence belief in God has over health life. We made minor revisions to fit the context of health to education. An example item is: ‘Whatever happens to my life is God’s will’. The Cronbach’s *α* was 0.84. Finally, learning achievement was measured by the current semester *grade point average* (GPA) on a scale of four.

### Control variables

As loneliness is closely related to demographic factors (e.g., Barreto et al., 2021; Page and Cole, 1991), we controlled for three control variables: age, degree, and gender. Each variable was coded as follows: gender (male = 0, female = 1), degree (undergraduate = 1, master’s student = 2), age (1 = <25 years old; 2 = 25–30 years old; 3 = >30 years old).

## Results

### Common method bias: concern, validity, and reliability

This study used a single data source for three variables (loneliness, engagement, and GLC), so it had the possibility of common method variance (CMV) (Fuller et al., 2016; Podsakoff et al., 2012) and common method bias (CMB). We took several steps to ensure that the data were free of CMV. First, we used an anonymous questionnaire to increase respondents’ objectivity and freedom to answer questions. Second, we used different type response measurement scales (agree-disagree; never-always). Third, we collected data in two phases to avoid respondent’s tendency to associate variables in the study (Podsakoff et al., 2012). During statistical analysis, we applied Harman’s one-factor test and exploratory factor analysis (EFA) to detect CMV and a full collinearity assessment approach with PLS-SEM (Kock et al., 2021; Kock, 2017).

Table 2 shows the single-factor results for all the constructs. The highest percentage of variance was 40.21% (engagement), followed by GLC (19.86%), and loneliness (14.81%), with a total variance of 74.88 percent, indicating that CMV is absent in this data. In the same vein, a complete collinearity test by alternately testing the dependent variable model found no VIF value >3.3, indicating that CMV in the data was not detected (Kock, 2017). Additionally, we used another procedure to minimize bias by separating the data collection time (time-lag) and the anonymity of the questionnaire (Podsakoff et al., 2012). Thus, concerns regarding CMV are reduced. Moreover, Table 2 shows that all variables met the requisite values for internal consistency (Cronbach’s *α* > 0.70), construct reliability (CR > 0.60), and AVE > 0.50, which are higher than the cut-off values recommended by Hair et al. (2010). For discriminant validity, the square root of the AVE of all variables was greater than the correlation between variables (see Table 3), indicating that discriminant validity is met (Fornell and Larcker, 1981).

**Table 2:** Reliability and validity of the study variables.

Construct	Number of items	CR	AVE	CA	%of variance
Loneliness	7	0.94	0.68	0.92	14.81
Engagement	9	0.96	0.75	0.93	40.21
God locus of control	6	0.96	0.79	0.84	19.86
Cumulative%					74.88
KMO and Bartlett’s Test					0.88

*n* = 324; *CR* construct reliability, *AVE* average variance extracted, *CA* Cronbach’s alpha, *KMO* Kaiser-Meyer-Olkin.

**Table 3:** Mean, SD, correlation and discriminant validity.

No	Variable	Mean	SD	1	2	3	4	5	6	7
1	Age	21.66	0.78	1						
2	Gender	1.64	0.48	−0.07	1					
3	Degree	1.27	0.45	0.06	0.04	1				
4	Loneliness	2.88	0.80	0.01	0.20**	−0.03	*(0.82)*			
5	Engagement	3.47	0.73	0.00	−0.08	−0.03	−0.38**	*(0.87)*		
6	Achievement	3.23	0.37	0.05	−0.14**	0.00	−0.29**	0.29**	*(0.89)*	
7	God locus of control	3.67	0.63	0.00	−0.07	0.05	−0.15**	0.12	0.19**	1

*n* = 324; **Correlation significant at 0.01; the square root of average variance extracted of discriminant validity is depicted diagonally in italics.

### Descriptive statistics

Table 3 presents the data’s descriptive statistics (mean, standard deviation) and bivariate correlation between variables. The correlation results show preliminary evidence of this hypothesis. Loneliness was proven to be negatively related to engagement (*r* = −0.38; *p* < 0.01) and academic achievement (*r* = −0.29; *p* < 0.01). As predicted, engagement was also positively related to achievement (*r* = −0.29; *p* < 0.01). The correlation of control variables indicates that gender plays an important role in engagement and achievement. For example, gender was negatively related to loneliness (*r* = −0.20; *p* < 0.01) and achievement (*r* = −0.14; *p* < 0.01).

### Hypothesis testing

The study adopted the PROCESS macro (Hayes, 2017) model 8 to test all hypotheses, including mediation and moderation analysis. The results in Table 4 display the hypothesis testing results. The first hypothesis proves the relationship between negative loneliness and engagement (*β* = −0.33; *t* = −6.84; *p* < 0.01). Loneliness was also found to be negatively associated with academic achievement (*β* = −0.08; *t* = −3.14; *p* < 0.01), and engagement was positively related to academic achievement (*β* = 0.11; *t* = 3.81; *p* < 0.01). Accordingly, H1–H3 were supported. H4 is the mediation hypothesis that examines engagement’s role in the relationship between loneliness and academic achievement. As shown in Table 3, the fourth hypothesis of the study was also proven (*β* = −0.004, SE = 0.01). We conducted bootstrapping estimation for additional testing on indirect effects and obtained results that supported the significance of previous findings (confidence interval [CI] using a 5000-bootstrap sample that did not include 0; CI was −0.06 and −0.02). Therefore, H4 was supported.

**Table 4:** Hypotheses evaluation (model 8 of Hayes’ PROCESS macro).

Predictors	*β*	SE	*T*	*p*	LLCI	ULCI
*Model 1: Outcome variable student engagement*						
Control variables						
Gender	0.00	0.08	−0.06	0.95	−0.16	0.15
Degree	−0.09	0.08	−1.03	0.30	−0.25	0.08
Age	0.01	0.05	0.19	0.85	−0.09	0.10
Main effect						
Loneliness	−0.33	0.05	−6.84	0.00	−0.43	−0.24
Interaction Effect 1	0.20	0.08	2.67	0.01	0.05	0.36
R-Sq	0.17					
*Model 2: Outcome variable academic achievement*						
Control variables						
Gender	−0.06	0.04	−1.43	0.15	−0.14	0.02
Degree	0.01	0.04	0.12	0.90	−0.08	0.09
Age	0.02	0.02	0.93	0.35	−0.03	0.07
Main effect						
Loneliness	−0.08	0.03	−3.14	0.00	−0.14	−0.03
Engagement	0.11	0.03	3.81	0.00	0.05	0.17
Interaction Effect 2	−0.08	0.04	−2.09	0.04	−0.16	0.00
R-Sq	0.16					

Bootstrapping results indirect effect						
	Effect	BootSE	BootLLCI	BootUL		
Indirect effect	−0.04	0.01	−0.06	−0.02		
Index of moderated mediation	0.02	0.01	0.00	0.05		

H5 and H6 are moderating hypotheses. Table 4 shows that the two interaction variables are statistically significant. First, interaction 1 (loneliness x the GLC) was significant in predicting student engagement (*β* = 0.20, *t* = 2.67, *p* < 0.01). Interaction 2, loneliness and the GLC, also proved significant (*β* = −0.08, *t* = −2.09, *p* < 0.05) in predicting academic achievement.

Table 5 also shows how GLC plays a role. First, in the relationship between loneliness and engagement, the GLC plays a positive role in reducing loneliness’ negative effect on engagement. As shown in Table 5, the effects ranges from −0.47 to −0.33 when GLC is low to high, respectively. These results indicate that when students have high levels of GLC, loneliness’ negative effect on engagement is reduced.

**Table 5:** Moderating effect of the God locus of control.

Conditional effects of the focal predictor at values of the moderator(s):						
		Effect	SE	*t*-value	LLCI	ULCI
Effect of LONE on ENG	Low GLC	−0.47	0.07	−6.89	−0.60	−0.34
moderated by GLC	High GLC	−0.33	0.05	−6.86	−0.43	−0.24
Effect of LONE on ACH	Low GLC	−0.03	0.04	−0.74	−0.10	0.05
moderated by GLC	High GLC	−0.14	0.04	−3.69	−0.21	−0.06

*LONE* loneliness, *GLC* God locus of control, *ENG* student engagement, *ACH* academic achievement.

**Fig. 2:**
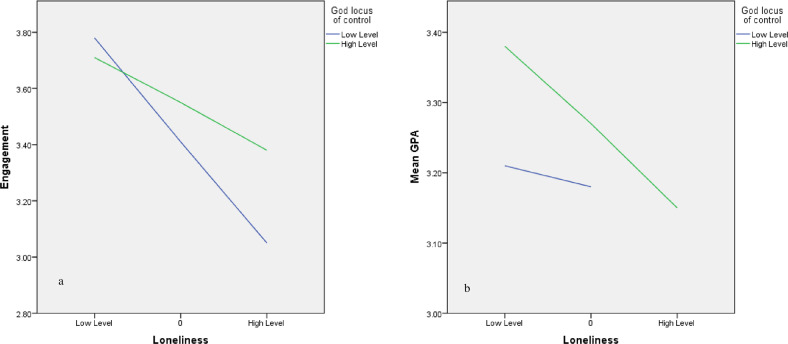
The moderating effect of GLC. **a** The moderating effect of the GLC on the relationship between loneliness and engagement; **b** the moderating effect of the GLC on the relationship between loneliness and academic achievement.

Second, in the relationship between loneliness and achievement, GLC plays a negative role by increasing loneliness’ negative effect on achievement. The effect of loneliness on achievement rises from −0.03 to −0.14 when GLC increases from a low to a high level. In contrast to interaction 1, this result indicates that higher GLC increases the effect of loneliness on achievement. Figure 2 illustrates these two effects.

## Discussion and conclusion

Loneliness is a widely studied phenomenon along with the increasing use of information technology in interaction. The phenomenon of loneliness in education has been studied for the last two decades. It has increased dramatically with the onset of online learning in all educational institutions to prevent the transmission of COVID-19. This study specifically explores the relationship between loneliness and student engagement and achievement among university students in Indonesia. We also add the GLC as a personality factor that acts as a boundary condition in the relationship. The current findings reveal unique findings and contribute to the field of distance education studies for educational and social stakeholders, which we discuss separately below.

### Theoretical implications

The present study focused on the relationship between loneliness, student engagement, and academic achievement. In addition, it emphasizes the GLC preferences as moderators of the relationship between loneliness, engagement, and academic achievement. The results here prove that loneliness has a negative effect on student engagement and academic achievement, thus confirming previous studies (i.e., Benner, 2011; Fan et al., 2021; Kaufmann and Vallade, 2020; Singh et al., 2021; Yalçın et al., 2020). Our study also found student engagement’s positive effect on academic achievement. The results of the study confirmed earlier studies (Bergdahl et al., 2020; Li et al., 2019; Pursel et al., 2016; Wang and Eccles, 2012; Yoon et al., 2018). Since loneliness’ adverse effects on student behavior have been widely proven, our study adds explicit insight to the models tested in several ways.

First, the study results showed that student engagement partially mediated the relationship between loneliness and academic achievement. Thus, we uncovered student engagement’s role as a unique psychological mechanism mediating the effect of loneliness on academic achievement. To our knowledge, this is the first study to examine these relationships. Our findings further supported the ETL theory (Cacioppo and Cacioppo, 2018): high levels of loneliness drive people to avoid social interactions (e.g., learning engagement). Moreover, our study extended the PNL model (Dormann et al., 2018) by exploring the indirect relationship of personal resources (loneliness) to achievement through learning engagement.

Second, the results here showed the GLC’s complex role in the relationship between loneliness, engagement, and academic achievement. It has already been established that the GLC itself is a personality trait associated with a person’s belief about the power of God in controlling their lives. Although derived from the concept of LoC (Rotter, 1966), which is well established in psychological research, the role of GLC as an internal or external source is debated. In this study, we uncovered a unique dual function of the GLC. In the relationship between loneliness and engagement, GLC acts as a positive religious coping behavior (Pargament, 2011) that neutralizes the destructive effects of loneliness on student engagement. Hence, the GLC may be equivalent to the sense of personal control (Spilka and Ladd, 2013), which may moderate the adverse effects of loneliness and decrease depression (Brewer and Olive, 2014; Simoni & Ortiz, 2003).

Our results showed that loneliness’ negative impact on engagement is reduced when individuals had high levels of the GLC. However, GLC played a different role in the relationship between loneliness and academic achievement. The higher the GLC level, the higher loneliness’ negative effect on achievement. In this model, we can argue that GLC is supported by the group of researchers who claim that GLC is associated with an external locus (e.g., Boyd and Wilcox, 2020a, 2020b; Welton et al., 1996; Zarzycka et al., 2021). However, we took a different approach in studying GLC’s role in student learning behavior. For example, Zarzycka et al. (2021) analyzed religion’s effect on students’ behavioral procrastination via God control, while our study analyzed its role as a moderator on the relationship between loneliness and student engagement as well as loneliness and achievement. Based on our findings, we conclude that deeply rooted belief in God’s control may weaken the negative effect of loneliness on student learning behavior. Students with a high level of perceived GLC may have external/alternative control (Spilka and Ladd, 2013), which causes them to be more resistant to loneliness’ destructive effects.

Finally, our study broadened the understanding of GLC’s complex role as an internal and external source. As an internal source, GLC increases self-efficacy, motivation, and determination and possesses high achievement motivation and engagement (Albert and Dahling, 2016; Boyd and Wilcox, 2020b; Cahyadi et al., 2021; Chukwuorji et al., 2018; Yang et al., 2017). The opposite result occurs if the GLC acts as an external source. This study found that GLC effectively acts as a positive religious coping behavior directly related to a decrease in depression (Brewer and Olive, 2014) caused by feelings of loneliness, but it does not have a direct effect on the actual achievement of student learning outcomes using GPA scores.

### Practical implications

This study’s results allow higher educational institutions and education policymakers to better understand the negative potential of implementing online learning. Administrators need to conduct special studies exploring signs of student loneliness through direct discussions or by using standardized instruments. Thus, schools must ensure that online teachers are well trained in technological literacy to solve technical and network problems and the situations/conditions of students directly affected by the pandemic. Classrooms must be designed for student communication and teachers should work towards fostering a healthy and positive environment for them. Teachers should also communicate in a manner that promotes student enthusiasm and reduces feelings of isolation and loneliness. Moreover, the GLC is vital in reducing the damaging effects of loneliness on learning engagement and student achievement. As a country that explicitly places trust in God’s power as national philosophy, we invite teachers in Indonesia to continue to promote students’ faith in God’s power to deal with the sense of isolation and loneliness experienced in online learning activities during the pandemic.

As interest in using online learning in education increases beyond the last 2 years, in anticipation of continued pandemic effects, we invite the whole society, including administrators, educators, and families in Indonesia, in particular, to be mindful of the potential student loneliness. Loneliness not only interferes with studies but also overall mental health. It would aid all stakeholders in better understanding the long-term impact on online learning modes, which have all the disadvantages associated with the lack of direct social interaction. Educational institutions may design models and methods that increase student activity in online classes, but the lack of non-verbal cues can create barriers to relational interaction. Although technology facilitates are convenient and offer excellent opportunities for students to flexibly access learning, it can trigger students’ feelings of loneliness owing to the lack of physical interaction with teachers and peers. Remote teaching can be an impersonal atmosphere, causing a lack of familiarity among students and an absence of sensitivity towards body language and non-verbal cues—a situation that does not provide a good environment for the development of students’ psychological and social abilities.

### Limitation and future directions

This study has some limitations. First, the participants were from over six universities from four provinces in Indonesia; thus, the results may not represent all the students in Indonesia or other countries. Future studies should explore similar topics in different regions and countries. Second, this study was carried out when the COVID-19 pandemic was ongoing, resulting in educational institutions implementing online learning. In an unnatural situation with social restrictions imposed by the government, the loneliness experienced by students may be related to this issue. Our study does not explicitly explain the causes of student loneliness. Future studies need to expand on the potential predictors of loneliness during pandemic and non-pandemic periods.

Third, the GLC in this study has two different faces: providing for internal sources/positive religious coping behavior in loneliness and engagement relationships and acting as an external source in loneliness and academic achievement relationships. We invite future studies to re-examine these findings in different cultures. Additionally, this study used a cross-sectional time-lag approach to minimize the CMV. However, the single data source we used remains of particular interest for future studies. Thus, we suggest that future studies use a longitudinal design and take data from different sources to ensure the causality of the relationship.

## Data Availability

All data analyzed are included in the paper.
